# The Get-Up! study: adiposity and blood pressure in Australian toddlers

**DOI:** 10.1097/j.pbj.0000000000000063

**Published:** 2020-07-17

**Authors:** Eduarda Sousa-Sá, Zhiguang Zhang, João R. Pereira, Sanne L.C. Veldman, Anthony D. Okely, Rute Santos

**Affiliations:** a Early Start, Faculty of Social Sciences, University of Wollongong; b Illawarra Health and Medical Research Institute, Wollongong, New South Wales, Australia; c Research Unit for Sport and Physical Activity, University of Coimbra, Coimbra; d Research Centre in Physical Activity, Health and Leisure, University of Porto, Porto, Portugal.

**Keywords:** adiposity, cardiovascular health, hypertension, pediatrics

## Abstract

**Background:**

Because the elevated blood pressure (BP) in childhood is strongly associated with overweight and is a risk factor for later cardiovascular disease, a need to comprehend the early development of BP and its association with overweight is needed. We assessed differences of BP by weight status in Australian toddlers.

**Methods:**

From the Get-Up! Study in Australia, this sample included 265 toddlers (136 boys), aged 19.6 ± 4.2 months. BP was measured with a digital vital signs monitor. Participants were categorized as nonoverweight and overweight according to the World Health Organization definition for body mass index (BMI). Physical activity was captured with activPAL accelerometers, during childcare hours. To test differences in BP between nonoverweight and overweight children, we performed an analysis of covariance adjusting for sex, age, physical activity, and socioeconomic status.

**Results:**

Children with overweight showed higher *z* systolic BP values (*P* = .042 for BMI and *P* = .023 for waist circumference) when compared to nonoverweight children. However, no differences were found for *z* diastolic BP levels, between overweight and nonoverweight children. After adjustments for potential confounders (socioeconomic status, physical activity, sex, and age), there were no significant differences in BP variables between BMI and waist circumference groups.

**Conclusions:**

No associations between adiposity and BP levels were found in this sample. The unadjusted results, however, showed that children with higher levels of adiposity (BMI and waist circumference) exhibited higher levels of BP. Additional research is needed to determine which environmental and genetic factors might contribute to pediatric hypertension, particularly among toddlers.

## Introduction

Hypertension is a significant cardiovascular disease (CVD) risk factor^1^ known to be established early in life.^2^ Regardless of CVD events often occur only during or after the fifth decade of life, pathophysiological and epidemiological evidence suggests that hypertension and the precursors of CVD initiate during childhood.^3^ The early onset of overweight trajectories has been associated with high blood pressure (BP) in late adolescence, in both boys and girls.^4^ In addition, some studies have shown that obesity is a key determinant to elevated BP during childhood and that weight reductions may have beneficial effects on BP, later in life.^5^ As obesity seems to be associated with high BP across all ages,^6^ it was frequently presumed in the last decades, that prevalence of hypertension had increased in youth.^7^ However, such trends have not been observed in all populations.^8^ Nevertheless, given the increase in the prevalence of overweight children in the last decades,^7^ the strong association between being overweight and high BP levels, as well as the hazards of having high BP during childhood, in later CVD,^8^ a better understanding of BP's early development and its relationship with overweight is necessary.

Data on the relationship between body mass index (BMI), waist circumference, and BP in toddlers are limited.^5^,^9^,^10^,^11^ The most recent study assessed 3186 children, aged 1 to 6 years, and found that mean systolic BP increased with BMI percentiles, but significant associations were not found with diastolic BP.^9^ Falkner et al^10^ showed that, based on BMI percentiles, overweight children, aged 2 to 5 years, had significantly higher mean diastolic and systolic BP values than those with normal weight. A Chinese study (1322 children, aged 0.1–6.9 years) showed that in children with obesity, an increase of 1 BMI unit was associated with, on average, an increase of 0.56 to 0.54 mm Hg in systolic and diastolic BP, respectively.^5^ A study with 2876 Australian children, aged 1 to 6 years, showed that per unit increase in BMI there was an increase in systolic and diastolic BP, at ages 1 and 3 years old.^11^ A limitation of these studies was that physical activity was not assessed and, therefore, statistical analyses did not account for this important variable. Indeed, it has been shown that low levels of physical activity are associated with the likelihood of being overweight and having high BP, in pediatric populations.^12^ In this context, our aim is to assess differences of BP by weight status in Australian toddlers, accounting for objectively measured physical activity levels.

## Methods

### Study design

Baseline data from the Get-Up! Study was analyzed. This study rationale and protocol can be found elsewhere.^13^ The Get-Up! Study is a 12-month, 2-arm parallel group cluster randomized controlled trial assessing the effects of reduced sitting time on toddlers’ cognitive development.

### Participants and protocol

The study included 335 healthy toddlers, aged between 11 and 29 months, from NSW, Australia. It was approved by the University of Wollongong's Human Research Ethics Committee (HE15/236) and conducted according to the Helsinki Declaration for Human Studies. Informed written consents were obtained from the children's parents or guardians, before collecting data (between March and August 2016).

A total of 265 toddlers (79.1%), aged 15 to 24 months (19.6 ± 4.2 months) had complete data on the variables of interest for this study (48% girls).

### Measures

*Anthropometrics:* Height, weight, and waist circumference were measured through standard procedures.^14^ Height was assessed to the nearest 0.1 cm with the child in bare or stocking feet, standing straight against a portable stadiometer (Seca 254 Hamburg, Germany) and weight was assessed to the nearest 0.1 kg, lightly dressed (without diapers), with a portable electronic weight scale (Seca 254 Hamburg, Germany). BMI was calculated as weight (kg)/height(m)^2^. Children were categorized as underweight, normal weight, or overweight or obese according to the World Health Organization age and sex specific criteria^15^ and divided into 2 groups: nonoverweight (underweight and normal weight children) and overweight (overweight and obese children), due to the small number of underweight and obese children of our sample.

Waist circumference was assessed at the top of the iliac crest with a nonelastic tape and *z* scores [*z* = (score-mean)/standard deviation (SD)] were calculated by age and sex, with participants being classified as nonoverweight (<1 SD of the *z* score) and overweight (≥1 SD of the *z* score).

*BP:* A digital vital signs monitor (WelchAllyn PROBP 3400 series, Skaneateles Falls, NY), which has been previously validated for this population,^16^ was used to assess BP in a quiet room, between 7 and 9 am. Assessments were taken from the right arm with a suitable cuff size^17^ with the participant in a sitting position. Two measurements were taken after 5 and 10 minutes of rest. Additional details on the assessment of outcomes of interest are elsewhere.^13^ *Z* scores by age and sex were computed for both systolic and diastolic BP, using standard procedures.

*Socioeconomic status (SES):* The Australian Socio-Economic Indexes for Areas 2011 (SEIFA – Index of Relative Socio-Economic Disadvantage)^18^ was used to assess family SES. SEIFA index starts from 1 (most disadvantaged), ends on 10 (least disadvantaged), and is based on the postcode. Participants were divided into 3 categories: low SES (deciles 1–3), middle SES (deciles 4–6), and high SES (deciles 7–10).

*Physical activity and sitting time:* Time spent in physical activity (stepping) and sitting were captured over a 1-week period during childcare hours, with an activPAL accelerometer. This device is a lightweight and small monitor, placed on the front of the upper thigh, which assess different postures (sitting, standing, lying). Concurrent and criterion validity of the activPAL for sitting time have been established for young children.^19^ The activPAL was fitted upon arrival and removed prior to the children leaving the childcare service in the afternoon. A log sheet to record each child's activPAL on and off times was given to educators, which was used to crossreference nonwear time and to manually eliminate nonwear time data. After collecting the monitors, data were downloaded and entered using activPAL software (v7.2.32). Fifteen-second epoch files were used to calculate the different postures and nonwear time for each participant, per day. Sequences of consecutive zero counts ≥20 minutes were deemed nonwear time and excluded from analyses. Naps taken while wearing activPAL were defined as nonwear time. To be considered valid data and included in the analyses, participants had to have at least ≥1 hour of wear time, on ≥3 days.

### Statistical analysis

IBM SPSS, version 24.0 (SPSS Inc, Chicago, IL) was used for data analyses. Descriptive analyses were presented as mean ± SD. Two-tailed Student *t* test or Mann-Whitney *U* test were performed to examine differences between boys and girls for continuous variables. Two-tailed Student *t* test and analysis of covariance (ANCOVA) were conducted (July 2018) to compare and to test differences, respectively, in BP variables between nonoverweight and overweight children (BMI and waist circumference groups). ANCOVA models were adjusted for SES, physical activity (minutes of stepping per hour of monitor wear time), sex, and age. The significance level for all tests was set at *P* < .05.

## Results

Participants’ characteristics are depicted in Table 1. In this sample, 0.4% was underweight, 19.8% was overweight, and 5% was obese. Boys had, on average, more minutes of stepping per hour than girls (*P* = .004). No significant differences were found for BMI, waist circumference, and BP between boys and girls. The majority (48%) of our sample falls within the lower decile of the SES, 34.3% belongs in the middle decile and only 17.7% on the high decile of SES.

**Table 1:**
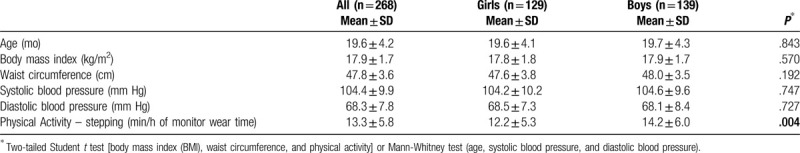
Participants characteristics

**Figure 1:**
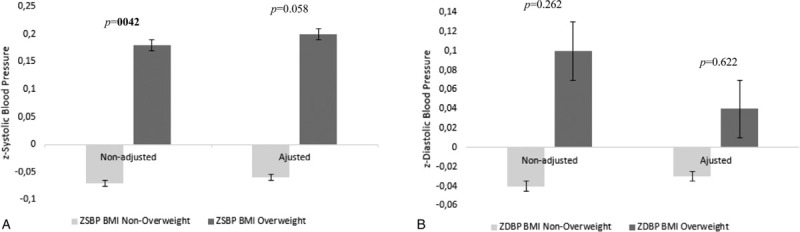
Analysis of covariance (ANCOVA) for systolic (A) and diastolic (B) blood pressure and BMI categories. BMI = body mass index.

**Figure 2:**
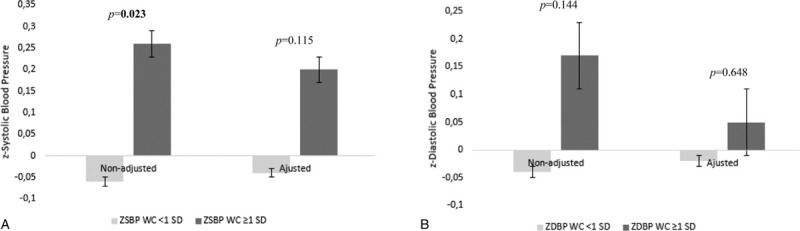
Analysis of covariance (ANCOVA) for systolic (A) and diastolic (B) blood pressure and waist circumferences *z* scores categories (nonoverweight <1SD of the waist circumference *z* score and overweight ≥1SD of the waist circumference *z* score). WC = waist circumference.

Figures 1 and 2 and supplementary Table S1, show the results of the *t* test and ANCOVA for BMI and waist circumference and BP values. *T* test results showed significant differences between BMI or waist circumference groups on *z* systolic BP levels, with the overweight groups showing higher *z* systolic BP values (*P* = .042 for BMI and *P* = .023 for waist circumference). No differences were found for *z* diastolic BP levels, between BMI groups. ANCOVA results showed no significant differences between BMI or waist circumference groups on *z* systolic BP or *z* diastolic BP levels (*P* > .05 for all) – Table S1.

## Discussion

After adjustments for potential confounders (SES, physical activity, sex, and age), there were no significant differences in BP variables between BMI and waist circumference groups. Despite the lack of significance, overweight children (classified by BMI or by waist circumference) showed higher values of systolic and diastolic BP *z* scores than nonoverweight children. Our results are, therefore, comparable to previous studies. An Australian study with 2876 children aged 1 to 6 years, found that per unit increase in BMI there was also an increase in BP; an average of 1.4 and 0.7 mm Hg for systolic and diastolic BP increase, respectively.^11^ This was also seen in a Chinese study comprising 1322 children aged 0.1 to 6.9 years.^5^ The authors showed that 19.4% of the obese children had hypertension. Likewise, a study with 18,618 American children aged 2 to 19 years, showed that higher BMIs were positively associated with BP in toddlers and preschoolers.^10^ A study with 3186 Iranian children aged 1 to 6 years, found evidence that increasing BMI is associated with increased systolic BP but no association was found with diastolic BP,^9^ which is similar to our results found in the unadjusted models.

A recent study showed that greater weight gains, particularly between the ages of 12 to 24 and 24 to 36 months, were associated with higher BP at the age of 3 years.^20^ However, another investigation found that stronger effect sizes for weight gain at later ages, occur closer to the age of 5.^21^ Considering these findings, as the age of our sample falls between 15 and 24 months and the cross-sectional nature of our analysis, this sample might be too young to show any significant predictive differences for elevated BP, at this stage. Nevertheless, the cumulative effects over time of higher levels of adiposity in our sample might be an important predictor for the future development of high BP.

Nowson et al^20^ found that greater gains in abdominal circumference between 0 and 6 and 24 and 36 months were associated with higher BP at 36 months (*P* < .001). Unlike BMI, waist circumference can provide better information on the patterns of fat distribution, particularly abdominal obesity.^22^ Waist circumference is, therefore, important for metabolic risk stratification. Indeed, fat distribution as a risk factor for elevated BP is well-documented, for central and peripheral fat.^23^ Undeniably, central adiposity has a stronger role than total adiposity in the development of pediatric hypertension. This is supported by several studies suggesting that waist circumference is a superior and more reliable predictor of BP when compared to BMI, among both normal weight and overweight children.^22^,^24^

Nevertheless, the relationship between central fat deposition with BP is not yet clear, in very young children.^9^ Some data indicated that rapid abdominal weight gain during infancy (0–6 months), but not during early childhood (3–6 years), was associated with a higher metabolic risk score at age 17 years, but the association between waist circumference and BP was not statistically significant.^25^

However, results regarding waist circumference in very young children should be viewed with caution as, unlike what happens with adolescents, abdominal circumference tends to decrease between the ages of 24 and 36 months.^20^ Thereby, increases in waist circumference during this period might be considered as an early marker for metabolic risk in later life, particularly if the effect of abdominal circumference increments amplifies the BP levels as age goes by.^20^ Indeed, it was expected to fail in finding significant associations between adiposity and BP in our sample of healthy toddlers, as the deleterious effect of excess of adiposity on BP is likely to manifest over time, as children get older.

Because our sample comprised a low number of overweight and obese children, we assume that this might have contributed greatly for our lack of statistically significant results, as it is known that overweight is a common risk factor for the development of high BP.^6^ Although we did not find significant results in the adjusted models, it is important to monitor BP levels and its relationship with weight early in life, as both weight and BP are known to track over life. The relationship between BP and cardiovascular risk is continuous, consistent, and independent of other risk factors.^26^ Growing data show that constant elevated BP in children leads to cardiovascular alterations,^27^ tracking from childhood through adolescence and into adulthood,^27^ in close association with weight,^28^ weight gain between 1 and 5 years^29^ and 2 to 4 years of age.^30^ This BP tracking is the most important argument for concerning with elevated BP early in life^31^; therefore, prevention and treatment of elevated BP early in life can result in a lifelong reduction of BP and of its associated conditions.

Most of the studies found in toddlers did not assess physical activity and, therefore the statistical analysis of those studies was not adjusted for this variable.^5^,^9^,^10^,^11^ Physical activity is well recognized as a preventive method for weight gain and high BP, in young children.^32^ Thus, our study makes an important contribute by adding physical activity to the confounding variables, as well as, assessing it objectively.

Also important are the specific cut-off values for BP, as no outcome-based definition of hypertension in toddlers is available. It could be argued that cut-off BP values to assess hypertension among toddlers should be based on distributions found in the target populations. Such cut-off values have been proposed for children in Italy and in the UK, but not for Australia. For this reason, we chose to use BP *z* scores in our analysis.

Strengths of our study include the unique young age of the participants and the use of valid and reliable tools to assess adiposity, BP, and physical activity. We used an oscillometric device to assess BP as they offer several advantages over auscultation. They correlate closely with intra-arterial pressures and the mean error is smaller than with the auscultatory method. Not just BMI but also one other measure of adiposity was used (waist circumference), better indicating fat distribution than BMI. Also, physical activity was assessed objectively.

Limitations to consider include the cross-sectional design, which prevents us to establish causality and the fact that we did not contemplate other potential confounders in our analysis, such as dietary intake, which would clarify the associations we observed. We acknowledge that we only assessed physical activity during childcare hours. Nevertheless, a recent review and meta-analysis looking at prevalence of objectively measured sedentary behavior in the early years showed that no significant differences were found between sedentary time while at childcare and nonchildcare hours.^33^ The only study included in that review using ActivPals did not analyze the difference between sedentary time while at childcare versus non-childcare hours.^34^ Moreover, the included toddlers’ samples were considerably older than ours, on average 6 months.^35^,^36^,^37^ Only one of them looked at sedentary time while at childcare and noticed that childcare attendance was only found to have a statistically significant effect on participants’ rates of light physical activity, assessed with an accelerometer.^37^ However, a study with 149 Canadian toddlers that objectively assessed sedentary time showed that toddlers engaged in approximately 6 min/day less sedentary time while at childcare when compared to those in parental care.^38^ Therefore, it is difficult to make assumptions regarding a potential impact of these periods in the present study. Also, at this young age, abdominal circumference may only represent a gross measure of central fat deposition and differences between individuals may represent genetically determined differences in physique. Moreover, our sample was not nationally representative and, thus, our results may not be generalizable to the Australian population. Although we assessed BP twice, this measure remains subject to variability originating from stress and personality factors and this may create measurement bias.

## Conclusions

In summary, although there is lack of statistical significance in our study, our results show that children with higher levels of adiposity (BMI and waist circumference) exhibit higher levels of BP. However, after adjustments for potential confounders, including physical activity, there was no significant association between adiposity and BP. Additional longitudinal studies looking at the association between adiposity and BP in toddlers might increase our understanding on how these 2 variables are connected and influence cardiovascular health later in life. Additional research is needed to determine which environmental and genetic factors might contribute to pediatric hypertension, particularly among toddlers.

## Acknowledgments

Assistance with the study: Alison McKinnon

Financial support and sponsorship: Sousa-Sá and Pereira have PhD Scholarships from the University of Wollongong; Zhang has a PhD scholarship from the China Scholarship Council and an International Postgraduate Tuition Award from University of Wollongong; Santos was supported by a Discovery Early Career Research Award from the Australian Research Council (DE150101921).

## Conflicts of interest

None.

## Supplementary Material

Supplemental Digital Content
