# Supplementary material for: The Get-Up! study: adiposity and blood pressure in Australian toddlers
Source: Porto Biomed J. 2020 Jul 17;5(4):e063. doi: 10.1097/j.pbj.0000000000000063 (PMC7386441; doi:10.1097/j.pbj.0000000000000063)
Supplement: Supplemental Digital Content [file pj9-5-e063-s001.doc]

**Table S1:** Differences in systolic blood pressure and diastolic blood pressure between non-overweight and overweight children

| **Variable** |  | ***t*-test** | | **ANCOVA^a^** | |
| --- | --- | --- | --- | --- | --- |
| Systolic BP *z*-scores | **BMI** | **Mean ± SE** | **F and *p* values** | **Mean ± SE** | **F and *p* values** |
|  | Non-overweight | -0.07 ± 0.07 | T= 4.182 | -0.06 ± 0.07 | F= 1.592 |
|  | Overweight | 0.18 ± 0.1 | **0.042** | 0.20 ± 0.12 | 0.058 |
| Diastolic BP *z*-scores |  |  |  |  |  |
|  | Non-overweight | -0.04 ± 0.06 | T= 1.263 | -0.03 ± 0.14 | F= 0.244 |
|  | Overweight | 0.10 ± 0.1 | 0.262 | 0.04 ± 0.14 | 0.622 |
|  |  |  |  |  |  |
| **Variable** |  | ***t*-test** | | **ANCOVA^a^** | |
| Systolic BP *z*-scores | **Waist circumference (*z*-scores)** | **Mean ± SE** | **F and *p* values** | **Mean ± SE** | **F and *p* values** |
|  | <1 SD | -0.06 ± 0.06 | T= 5.199 | -0.04 ± 0.15 | F= 2.498 |
|  | ≥ 1 SD | 0.26 ± 0.12 | **0.023** | 0.20 ± 0.15 | 0.115 |
| Diastolic BP *z*-scores |  |  |  |  |  |
|  | <1 SD | -0.04 ± 0.06 | T= 2.140 | -0.02 ± 0.15 | F= 0.209 |
|  | ≥ 1 SD | 0.17 ± 0.13 | 0.144 | 0.05 ± 0.15 | 0.648 |

^a^ Adjusted for socio-economic index, physical activity (stepping min/hour of wear time), sex and age.

SE: Standard error
